# Flaxseed Lignan Complex Administration in Older Human Type 2 Diabetics Manages Central Obesity and Prothrombosis—An Invitation to Further Investigation into Polypharmacy Reduction

**DOI:** 10.1155/2012/585170

**Published:** 2012-10-04

**Authors:** D. E. Barre, K. A. Mizier-Barre, E. Stelmach, J. Hobson, O. Griscti, A. Rudiuk, D. Muthuthevar

**Affiliations:** ^1^Department of Health Studies, Cape Breton University, P.O. Box 5300, Sydney, NS, Canada B1P 6L2; ^2^Department of Biology, Cape Breton University, P.O. Box 5300, Sydney, NS, Canada B1P 6L2; ^3^Department of Biology, Kwantlen Polytechnic University, 8771 Lansdowne Road, Richmond, BC, Canada V6X 3V8; ^4^School of Nursing, Cape Breton University, P.O. Box 5300, Sydney, NS, Canada B1P 6L2; ^5^Faculty of Medicine, Saint James School of Medicine, Bonaire, The Netherlands

## Abstract

*Aim*. Animal and human study evidence supports the hypothesis that flaxseed lignan complex (FLC) at a dose of 600 mg secoisolariciresinol diglucoside (SDG)/day for three months would combat hyperglycaemia, dyslipidemia, blood pressure, central obesity, prothrombotic state, inflammation, and low density lipoprotein (LDL) oxidation. *Methods*. Sixteen type 2 diabetic patients completed this double-blind, randomised crossover placebo-controlled study. A univariate repeated measures analysis of covariance (significance *P* < 0.05) was followed by a mixed linear model effects analysis corrected for multiple comparisons (MCC). *Results*. Prior to MCC, FLC caused decreased fasting plasma glucose, A1c, inflammation (c-reactive protein (CRP) and interleukin-6 (IL-6)), and increased bleeding time. After correction for multiple comparisons, FLC induced a statistically significant increase in bleeding time and smaller waist circumference gain. No treatment effect occurred in the other variables before or after adjustment. *Conclusions*. It is concluded that FLC significantly increased bleeding time thus reducing the prothrombotic state, reduced central obesity gain as measured by waist circumference, and did not affect significantly the other dependent variables measured after adjustment for multiple comparisons. These findings, not yet published in human type 2 diabetes, suggest that this FLC dose over at least three months, may, subject to further investigation, reduce polypharmacy.

## 1. Introduction

Type 2 diabetics face numerous clinical features. Chief among these are seven: central obesity, hyperglycaemia, dyslipidemia, hypertension, inflammation, LDL oxidation, and the prothombotic state. The sequelae of these features, atherosclerotic-driven myocardial infarction and stroke [1, 2], are the major causes of disability and death in type 2 diabetes patients [3]. Polypharmacy (concurrent use of numerous drugs) currently used to combat these seven issues is of serious concern [4]. Ideally, one or a smaller number of drugs would safely and efficaciously address all seven issues.

Type 2 diabetes is diagnosed when there is a fasting plasma glucose of ≥7.0 mmol/L or, as the result of a 2 hour after oral glucose tolerance test (or at any random time of the day), a blood glucose level of ≥11.1 mmol/L. These numbers must be combined with reduced insulin sensitivity and/or blood plasma insulin levels to yield a diagnosis of type 2 diabetes. Central obesity is associated with type 2 diabetes and contributes to the other six issues. Central obesity may be defined as a waist circumference greater than 94 cm in males and 80 cm in females of Europid origin, the patients of the current study. Central obesity leads to the sequential cascade of hyperglycaemia, dyslipidemia and hypertension, oxidised low density lipoprotein (LDL), inflammation, plaque rupture, and thrombosis [2]. Obesity causes hyperglycaemia [5]. Hyperglycaemia (fasting ≥7.0 mmol/L) contributes to dyslipidemia as reflected in low plasma high density lipoprotein-cholesterol (HDL-c) levels (<1.0 mmol/L (males), 1.3 mmol/L (females)), increased triglycerides (>1.5 mmol/L), total cholesterol (TC) : HDL-c ratio (<4.0), small dense (sd)-LDL-c (≥1.1 mol/L), and sometimes elevated cholesterol (>5.2 mmol/L) and low density lipoprotein-cholesterol (LDL-c) concentrations (>2.0 mmol/L) [2], all of which contribute to plaque formation. Dyslipidemia and hyperglycaemia lead to hypertension in type 2 diabetics, defined as ≥130/80 mm Hg [2, 5]. Hypertension may lead to plaque rupture that contributes to the prothrombotic state [2], reflected in shortened bleeding times (under 5 minutes) [6]. Reduced antioxidant defences in type 2 diabetes [2] result in increased oxidised LDL which promotes increased synthesis of proinflammatory cytokines interleukin-6 (IL-6) and tumour necrosis factor alpha (TNF-*α*), major and minor modulators, respectively, of blood plasma c-reactive protein (CRP) levels [7]. Elevated CRP (levels of greater than 2 mg/L of plasma CRP) increases blood glucose levels (for review see [8]) with the cascading impact of hyperglycaemia illustrated above. 

Flaxseed lignan complex (FLC) containing secoisolariciresinol diglucoside (SDG) (and its colonic bacteria metabolites, enterolactone (ENL), and enterodiol (ED)), cinnamic acids (CA), and 3-hydroxy-3-methyl glutaric acid (HMGA) has been proposed collectively as agents that may manage all seven issues and hence potentially moderate their sequelae. FLC lowers blood plasma glucose [9] and A1c [10] in humans. Dietary lignans (e.g., SDG) have been suggested to control waist circumference [11] in humans and to decrease systolic and diastolic pressure in rats [12]. Kreijkamp-Kaspers et al. [13] showed that unspecified dietary lignans decrease blood pressure in humans. SDG has been shown to decrease triglycerides in rats [14] and increase HDL-c and lower cholesterol in rabbits [15]. ED and ENL have been proposed to manage oxidation [16] while SDG ingestion reduces inflammation [17]. CA are antioxidants [18] that lower platelet reactivity [19], and hence the prothrombotic state. In animal studies only, CA lowers cholesterol [20] (a feature shared with HMGA in humans) [21].

 The objective of the study was to test the hypothesis that flaxseed would safely combat the seven issues (i.e., reduce hyperglycaemia, dyslipidemia, hypertension, central obesity, inflammation, LDL oxidation, and the prothombotic state) thus potentially offering a means of reducing polypharmacy. The parameters collectively and singly (central obesity and prothrombotic tendency) are a novel investigation of FLC in type 2 diabetes management.

## 2. Materials and Methods

### 2.1. Study Design and Participants

Twenty-four subjects who were recruited via a local newspaper advertisement met the inclusion criteria. The inclusion criteria were a willingness to follow study protocol, 55 years of age or older, being postmenopausal (no menstruation for at least one year), not on insulin or changing exercise patterns, and healthy aside from type 2 diabetes (including no liver or kidney disease), and absence of change of medication(s) (dose and type) throughout the study. These inclusion criteria also applied for 3 months prior to the first visit. During the course of the study, eight patients dropped (4 moved away, two were no longer interested in the study, one developed rapidly and deeply fluctuating blood glucose (just before the start of the study), and one developed pruritis). Sixteen subjects completed the study. Ethical approval was granted by the Cape Breton University Human Ethics Committee and all subjects signed informed consent prior to the start of the study.

The study was a double-blind randomised crossover placebo-controlled study and consisted of four visits each 3 months apart. Patients were informed of the details of the study, consented, and asked three months prior to visit one and throughout the course of the study to refrain from consuming flaxseed or any of its components. All patients were asked to maintain their usual dietary and exercise patterns and stay on all over the counter and prescribed medications (except for flaxseed or any of its components) unless directed otherwise by their physician(s). At visits 1 to 4 inclusive patients had blood drawn from the antecubital vein and had their weight, height, waist circumference, bleeding time, and blood pressure determined. Patients then consumed either FLC (4 capsules—600 mg total SDG/day) or 4 identical looking placebo capsules/day for 3 months. FLC contained 32.9% SDG, 13.9 percent CA, 11.8% protein, 10.0% HMGA, 3.5% fat, 3.3% moisture, and 1% ash. From visit 2 to visit 3 no FLC or placebo was administered. At visit 3, patients were switched to whichever intervention to which they had not been exposed from visits 1 to 2. At visits 2, 3, and 4 patients turned in all dietary, smoking, exercise, and medication records for the previous 3-month period (patients were asked at visit 1 to keep such records for three consecutive days every two weeks during the entire trial).

### 2.2. Laboratory Measurements

The following measures were carried out ((kits and manufacturers in brackets) following manufacturers' directions): waist circumference [22], glucose (Wako Glucose C2, Wako USA, Richmond, VA, USA), A1c [23], triglycerides (L-type triglyceride M, Wako USA, Richmond, VA, USA), cholesterol (cholesterol E kit (WakoUSA, Richmond, VA, USA), HDL-c (precipitation via Quantolip HDL, Technoclone Vienna, Austria and cholesterol via cholesterol E kit-WakoUSA, Richmond, VA, USA), LDL-c (L- type LDL-c, WakoUSA, Richmond, VA, USA), sd LDL-c [24], LDL oxidation (oxidised LDL ELISA kit (Mercodia, Winston-Salem, North Carolina, USA), CRP (c-reactive protein (human EIA kit), Cayman Chemical, Ann Arbor, MI, USA), IL-6 and TNF-*α* (Quantikine HS elisa kits for human IL-6 and TNF-*α*, R and D systems, Minneapolis, MN, USA), and bleeding times (Surgicutt, ITC, Edison, NJ, USA). Compliance was assessed by blood plasma enterolactone levels (enterlactone EIA kit, Cayman Chemical, Ann Arbor, MI, USA). All blood plasma measures were carried out in duplicate and the values were averaged to give a mean value for each subject at each sampling time point for use in the statistical analysis.

### 2.3. Statistical Analysis

Data is expressed as mean ± SEM. Initially, each dependent variable (i.e., univariate) was assessed by itself using a repeated measures analysis of covariance with subjects as the random factor and age, sex, and order of treatment as covariates. Following that a repeated measures linear mixed model analysis of covariance was performed, using subjects as the random factor and age, sex, and order of treatment as covariates, with adjustment for multiple comparisons (i.e., over multiple dependent variables). Treatment effects are reported as FLC versus placebo. Power was calculated for a 0.4% change in A1c with a SD of 0.3%, a 1-*β* of 90%, a two tailed *α* = 0.05, and, consistent with other clinical trial work done by the lead author, a 33% dropout rate. This dropout rate required 16 patients to finish the study with an initial enrolment of 24 patients.

## 3. Results and Discussion

### 3.1. Baseline Characteristics

Baseline characteristics of the sixteen patients who completed the study are found in Table 1. Subjects were obese, hyperglycemic, dyslipidemic, hypertensive, and prothrombotic (as measured by reduced bleeding time) had elevated oxidised LDL and inflammation as measured by CRP, IL-6, and TNF-*α*. All comparisons reported are for treatment versus placebo.  Table 2 shows the impact of FLC on hyperglycaemia, dyslipidaemia, blood pressure, bleeding time, oxidised LDL, inflammation, and waist circumference.

### 3.2. Effects of FLC on Hyperglycaemia, Dyslipidaemia, Blood Pressure, Bleeding Time, Oxidised LDL, Inflammation, and Waist Circumference

Table 2 shows the impact of FLC on hyperglycaemia, dyslipidaemia, blood pressure, bleeding time, oxidised LDL, inflammation, and waist circumference.

Fasting blood plasma glucose fell 0.6 mmol upon FLC administration and only 0.3 mmol/L in the placebo arm. Correspondingly, A1c dropped 0.3 percent in the FLC arm but showed no change as a result of placebo administration. Both blood glucose and A1c fell significantly as a result of FLC administration compared to placebo for the univariate analysis but not when controlled for multiple comparisons. 

None of the blood lipid/lipoprotein parameters were changed significantly upon FLC administration relative to placebo consumption. 

Univariate analysis and adjustment for multiple comparisons revealed that neither systolic or diastolic pressures changed significantly as the result of FLC versus placebo consumption.

Placebo consumption patients gained 2.1 cm in waist circumference over the three-month period and only 0.6 cm upon FLC exposure. In the FLC group waist circumference started at 99.5 cm and finished at 100.1 cm while in the placebo group patients began with a waist circumference of 99.4 cm and ended at 101.5 cm. Adjustments for multiple comparisons indicated better waist circumference management for the patients consuming FLC relative to the placebo arm.

Bleeding time was significantly and very substantially increased by 50 seconds (224.4 to 274.6 seconds) as a result of FLC administration compared to 6 seconds (227 to 233 seconds) for the placebo consumers (Table 2). This held true for both the univariate and after adjustment for multiple comparisons analysis.

FLC caused a significant reduction in inflammation as measured by univariate analysis of reductions in CRP and the main modulator of CRP formation, IL-6 but not its minor modulator TNF-*α* when controlled for multiple comparisons. CRP went from 2.4 to 1.9 mG/L of plasma in the FLC consumers and did not change from 2.7 mG/L in the placebo group. IL-6 levels dropped from 4.1 to 3.6 pG/mL while the IL-6 measured at 4.3 pG/mL initially in the placebo group ended at 5.4 pG/mL. TNF-*α* was consistent at 1.1 pG/mL across the board in the FLC and placebo groups. However, CRP, IL-6, and TNF-*α* levels were not significantly different as the result of adjusting for multiple comparisons when FLC was compared to placebo. 

There was no FLC treatment impact on LDL oxidation relative to placebo when assessed by univariate analysis. Over the three-month period there were drops in LDL oxidation levels in both arms of the study from 52.2 to 45.2 (FLC) and 57.0 to 52.8 U/L (placebo).

Compliance was assessed by determining blood plasma enterolactone concentrations. A one-way analysis of variance indicated that FLC induced a statistically significant (*P* < 0.05) rise in ENL levels from 1039 ± 175 pg/mL to 4638 ± 618 pg/mL while in the placebo group enterolactone levels ranged from 1036 ± 158 (start of placebo) to 870 ± 141 pg/mL (end of placebo). There was no statistically significant difference between any combination of pre-FLC, preplacebo and postplacebo blood plasma enterolactone levels while post-FLC values were statistically significantly different (*P* < 0.05). Subjects as a population complied well with the prescribed FLC dosage as indicated by the statistically significant rise in enterolactone levels not seen with the placebo. 

As shown in Table 3, there was no statistically significant change in any of the following patterns. No patient smoked. No patient completing the study changed their prescribed or over the counter medication regimen (dose or medication) during the course of the study. Diet and exercise patterns remained the same throughout the three periods (visits one to two, visits two to three, and visits three to four). 

Univariate analysis revealed that both blood glucose and A1c fell significantly as the result of FLC administration compared to placebo. The fasting glucose drop has been observed using FLC in hypercholesterolaemic but otherwise healthy subjects using 600 mg/d SDG at 6 and 8 weeks [9]. Pan et al. (2007 [10]) did not show a drop in blood glucose in type 2 diabetics using FLC (360 mg SDG/day) for 12 weeks but did show a very mild drop in A1c levels (decrease of 0.11 A1c percentage points). The higher dose in the current study produced a more dramatic drop in A1c (0.3 A1c percentage points) consistent with the significant drop seen in fasting glucose levels. However, upon controlling for multiple comparisons these differences disappeared. Larger numbers of subjects may reveal statistically significant drops in both these parameters corrected for multiple comparisons.

None of the blood lipid parameters were changed. This is consistent with other though lower SDG dose FLC studies in human type 2 diabetics [10] and in healthy normolipidaemic postmenopausal women receiving FLC (500 mg SDG/day) in the form of muffins for 6 weeks relative to placebo [25]. However, Zhang et al. (2008 [9]) administering a dose of 600 mg/day SDG for 8 weeks found a decrease relative to placebo in cholesterol, LDL-c, and the total cholesterol : HDL-c ratio in humans with hypercholesterolaemia and hypertriglyceridaemia but who were otherwise healthy. Fukumitsu et al. [26] found no change in any lipid parameters with 20 mg/day SDG administered in the form of flaxseed lignan extract for 12 weeks relative to placebo and only a drop in the LDL-c-to-HDLc ratio relative to placebo. sd-LDLc and HDLc normally change in the same and opposite directions, respectively, as triglycerides. As there was no change in triglycerides it is not surprising that sd-LDLc and HDLc did not change as the result of FLC ingestion. Consequently dyslipidemia-driven atherosclerosis was not changed. Cholesterol and LDL-c levels were normal and generally nutraceuticals are more successful with managing elevated levels of these parameters.

In terms of systolic and diastolic blood pressures, FLC and placebo both showed a downward trend from their introduction to finish of application. Neither systolic nor diastolic pressures changed similar to the findings of Pan et al. (2007 [10]) in type 2 diabetics despite a higher FLC dose in the current study. The pre-FLC/placebo systolic pressures were somewhat higher and the diastolic pressures marginally lower in the Pan et al. [10] study compared to the current study. It may be that higher pressures are required initially before an FLC treatment effect can be seen. Cornish et al. (2009 [27]) showed, in a study subset, that those with metabolic syndrome with diastolic pressures averaging 88.7 mm Hg, the FLC dose of 543 mg/day for 6 months yielded a decrease in diastolic pressure. Regardless of metabolic syndrome status males but not females showed a decrease in diastolic pressure (initial level for diastolic pressure in males was 86 mm Hg) [27]. In the current study, univariate analysis revealed a *P* value of 0.115 for the drop in the diastolic pressure but like the results of Cornish et al. (2009 [27]), there was nowhere close to significance of any change in systolic pressures (again, systolic pressures were similar between the current study and Cornish et al. 2009 [27]). Thus, greater numbers of subjects with elevated diastolic pressures in a future study might yield a statistically significant drop in that pressure in type 2 diabetics. 

FLC produced a significantly different management of waist circumference in both univariate and multiple comparisons analysis. This is the first reported finding of such and may be due to lignan consumption in general being correlated with reduced waist circumferences [27]. Fukumitsu et al. [28] indicated that SDG lowered fat gain in mice consistent with the observation herein of lowered waist circumference in the FLC compared to placebo arm. 

Decreased bleeding time is associated with the prothrombotic state seen in type 2 diabetics [29]. Bleeding time is negatively associated with thromboxane B_2_ formation during whole blood platelet aggregation and in vitro platelet aggregation in type 2 diabetics [30]. Thrombosis risk is enhanced in type 2 diabetes due to hyperglycaemia [30], dyslipidaemia and increased inflammation (for review see [31]). Interestingly, in the current study, when univariate analysis was performed, hyperglycaemia and inflammation (as measured by CRP reduction) were reduced while dyslipidaemia was not changed. This implies that the impact on prothrombotic reduction was due to a reduction in hyperglycaemia and inflammation. As thrombus/embolus formation frequently induces myocardial infarction and stroke, the major causes of death and disability in type 2 diabetics, one might suggest that there is potential benefit in the reduction of the prothrombotic state by FLC; certainly this is worthy of further investigation. In the current study, the significantly increased bleeding time and resultant reduction in the prothrombotic state were as a result of FLC administration, a novel observation. This may have been due to the presence of cinnamic acids which have been suggested to decrease platelet reactivity [19]. Enterolactone may also reduce platelet glycoprotein IIb/IIIa expression [32] which would lower platelet aggregation. There appear to be no published reports on HMGA and platelet reactivity. 

FLC caused a significant reduction in inflammation as measured by reductions in CRP and the main modulator of CRP formation, IL-6 when only univariate analysis was used. The drop in IL-6 in the current study using univariate analysis contrasts with an absence of change with a similarly dosed and statistically analysed older but healthy adult population [31]. The absence of change in TNF-*α* is consistent with Cornish et al. (2009 [27]) with both studies using similar univariate statistical analyses. Hallund et al. (2008 [33]) showed no changes in CRP, IL-6, or TNF-*α* as a result of FLC (500 mg SDG/day) for 6 weeks to postmenopausal women using a similar univariate analysis (unadjusted for multiple comparisons). Pan et al. (2009 [34]) administering FLC (360 mg SDG/day) to type 2 diabetics for 12 weeks in a study of similar design to the current study found that CRP level rises were reduced relative to placebo and there were no treatment changes in IL-6 or TNF-*α* relative to placebo (again uncorrected for multiple comparisons). It may also be that the somewhat higher CRP and IL-6 levels in the current study as compared to those reported by Pan et al. (2009 [34]) caused their amenability to be greater. While these CRP and IL-6 statistically significant differences disappeared due to adjustment for multiple comparisons it should be pointed out that a number of studies have shown that FLCs reduce CRP. This was the first study to show that at doses higher than 300 mg SDG/day it is the IL-6 and not the TNF-*α* modulating the blood plasma glucose concentration reduction in type 2 diabetics as a result of FLC consumption. IL-6 and TNF-*α* are major and minor factors, respectively, that modulate blood plasma CRP levels [7]. Plasma CRP levels partially regulate plasma glucose concentrations (for review see [8]) and represent an index of plaque stability [35]. The reduction in CRP by univariate analysis is consistent with reduced blood glucose levels and suggests better plaque stability. Better plaque stability reduces the prothrombotic state. 

Increased LDL oxidation as measured by the LDL oxidation ELISA used in this study is associated with cardiovascular disease and stroke risk [36, 37]. There was no treatment impact on LDL oxidation for the 16 patients who completed the trial. This is the first human FLC trial to look at this parameter. Thus, cardiovascular disease risk associated with oxidised LDL was not reduced using this FLC complex dose for 3 months in this population. Interestingly, Hallund et al. (2006 [25]) showed no change in serum lipoprotein oxidation lag time as a result of FLC (500 mg SDG/day) for 6 weeks to postmenopausal women. The Hallund et al. 2006 [25] study did not measure specifically oxidised LDL. Thus, in the current study population, an absence of change in oxidised LDL cannot contribute to the lowered cardiovascular disease and stroke risk suggested by increased bleeding time, as well as improved management of central obesity and potentially hyperglycaemia.

There was no statistically significant change in dietary patterns. No patient smoked. No patient completing the study changed their medication regimen (dose or medication) or absence thereof at any point during the study. Thus dietary patterns, smoking, medication, and physical activity patterns have not contributed to the results indicating that differences are due solely to the FLC.

## 4. Conclusions

It is concluded that this FLC given at a dose of 600 mg SDG/day for three months combats in a statistically significant fashion, in this study population, waist circumference (central obesity) gain, and the prothrombotic state and hence potentially the risk of myocardial infarction and stroke. It is important to note that these changes took place despite the consistent consumption of various medications concurrently used to combat these issues. However, in addition, much larger numbers of subjects may reveal decreases in glucose and A1c, CRP, and IL-6 levels using the same protocol with adjustments for multiple comparisons. Thus, this is the first published indication of the potential for any agent to reduce polypharmacy in terms of the combination of waist circumference and prothrombotic tendency management and to hint at the potential at simultaneous management of hyperglycaemia via reduced inflammation in terms of potentially reduced CRP and its major driver IL-6. It is also concluded that this FLC dose for the three-month timeframe in this population does not impact the blood pressure or TC : HDL-c ratio or any of its lipid/lipoprotein contributors to that ratio. However, it must be very strongly cautioned that this was a small study. A more definitive answer to the question as to whether this unique FLC will match or exceed the benefits of reduced vascular complications resulting from polypharmacy used to address the unique combination of hyperglycaemia, waist circumference, prothrombotic state, and inflammation remains to be determined from a much larger multicentre, longer term trial planned by this laboratory using the same protocol. Of course, ultimately only complete FLC substitution for such medications addressing hyperglycaemia, waist circumference, the prothrombotic state and inflammation will address the issue of potential reduced polypharmacy directed at these four issues; that trial will be carried out upon successful indications of such from the larger study mentioned immediately above. It is particularly intriguing that FLC appears to have the potential to reduce the inflammation contributing to plaque rupture and hence the prothrombotic tendency, the last chance to stop the cascade before myocardial infarction or stroke, all apparently with minimal side effects or other complications.

## Figures and Tables

**Table 1:** Patient characteristics upon entry.

Parameter	Value
Age (years)	66.2 ± 1.7
Waist circumference (cm)	99.3 ± 4.2
BMI (kg/m^2^)	31.2 ± 2.2
Glucose (mMol/L)	7.1 ± 0.8
A1c (%)	7.2 ± 1.2
HDL-c (mMol/L)	1.1 ± 0.1
LDL-c (mMol/L)	2.5 ± 0.1
Small dense LDL-c (mMol/L)	0.3 ± 0.04
Cholesterol (mMol/L)	4.6 ± 0.3
Triglycerides (mMol/L)	1.7 ± 0.2
BP: systolic (mm Hg)	137.4 ± 5.1
BP: diastolic (mm Hg)	83.3 ± 2.1
CRP (mG/L)	2.6 ± 1.2
IL-6 (pG/mL)	3.7 ± 1.9
TNF-*α* (pG/mL)	1.2 ± 0.2
Bleeding time (seconds)	219.2 ± 11.6
Oxidised LDL (U/L)	56.8 ± 4.6

Values reflect duplicate assays of each blood sample (volumes are for plasma). Values are rounded to the nearest tenth. Data (mean ± SEM) is for the 16 patients who completed the trial.

**Table 2:** Impact of flaxseed lignan complex on hyperglycaemia, dyslipidaemia, blood pressure, waist circumference, bleeding time, LDL oxidation, and inflammation.

	Lignan start	Lignan finish	Placebo start	Placebo finish	Treatment versus placebo (U/M)*
Glucose (mMol/L)	7.2 ± 0.4	6.6 ± 0.4	8.0 ± 0.8	7.7 ± 0.6	*P* = 0.035/0.188
A1c %	7.1 ± 0.3	6.8 ± 0.2	7.2 ± 0.3	7.2 ± 0.3	*P* = 0.042/0.2911
HDL-c (mMol/L)	1.0 ± 0.1	1.1 ± 0.2	1.1 ± 0.3	1.2 ± 0.1	*P* = 0.423/NP^+^
LDL-c (mMol/L)	2.5 ± 0.2	2.4 ± 0.2	2.4 ± 0.1	2.3 ± 0.1	*P* = 0.437/NP
sd-LDL-c (mMol/L)	0.3 ± 0.1	0.2 ± 0.03	0.3 ± 0.03	0.2 ± 0.03	*P* = 0.126/NP
Cholesterol (mMol/L)	4.4 ± 0.3	4.5 ± 0.3	4.6 ± 0.3	4.5 ± 0.3	*P* = 0.382/NP
Cholesterol/HDL-c ratio	4.4 ± 0.3	4.1 ± 0.5	4.2 ± 0.1	3.8 ± 0.3	*P* = 0.701/0.3446
Triglycerides (mMol/L)	1.7 ± 0.2	1.8 ± 0.3	2.0 ± 0.4	1.8 ± 0.2	*P* = 0.350/NP
B.P. systolic (mm Hg)	133.6 ± 4.8	124.4 ± 4.7	135.8 ± 4.3	123.8 ± 3.6	*P* = 0.805/0.620
B.P. diastolic (mm Hg)	82.1 ± 1.9	74.9 ± 3.2	84.5 ± 2.2	78.3 ± 2.1	*P* = 0.115/0.774
Waist (cm)	99.5 ± 4.0	100.1 ± 4.1	99.4 ± 4.0	101.5 ± 3.9	*P* = 0.127/0.0374
Bleeding time (seconds)	224.4 ± 11.7	274.6 ± 9.5	227.1 ± 10.3	233.2 ± 9.4	*P* = 0.001/0.005
LDL oxidation (U/L)	52.2 ± 5.2	45.2 ± 4.6	57.0 ± 5.8	52.8 ± 4.8	*P* = 0.729/0.319
CRP (mG/L)	2.4 ± 1.1	1.9 ± 0.7	2.7 ± 1.2	2.7 ± 1.1	*P* = 0.029/0.372
IL-6 (pG/mL)	4.1 ± 2.3	3.6 ± 1.7	4.3 ± 1.9	5.4 ± 2.2	*P* = 0.017/NP
TNF-*α* (pG/mL)	1.1 ± 0.2	1.1 ± 0.1	1.1 ± 0.2	1.1 ± 0.2	*P* = 0.676/NP

Values reflect duplicate assays of each blood plasma sample. Values are rounded to the nearest tenth/hundredth. Data (mean ± SEM) is for the 16 patients who completed the trial. The univariate data analysis was a repeated measures analysis of covariance (covariates: age, sex, and order of treatment) controlled for multiple comparisons. U/M*: univariate/multiple comparisons corrected via mixed linear effects model controlling for age, gender, and order of treatment. ^+^NP: not performed, the parameters driving the major outcomes were not part of the multiple comparisons adjustment.

**Table 3:** Dietary and exercise consistency throughout the study.

	Visits 1 to 2	Visits 2 to 3	Visits 3 to 4	Adjusted *P* ^M^
Calories consumed (per day)	2054.3 ± 189.6	2032.8 ± 175.6	2049.4 ± 239.7	0.8043
Total fat (g/day)	77.3 ± 9.0	72.4 ± 6.5	78.1 ± 9.3	0.8947
Total protein (g/day)	90.4 ± 5.0	87.8 ± 6.3	85.8 ± 6.9	0.3113
Total carbohydrates (g/day)	257.5 ± 28.3	270.5 ± 32.4	261.8 ± 41.1	0.8383
Calories spent during exercise	259.8 ± 87.4	247.8 ± 99.8	247.8 ± 62.0	0.8266

Values are rounded to the nearest tenth. Data (mean ± SEM) is for the 16 patients who completed the trial. M: multiple comparisons corrected via mixed linear effects model controlling for age, gender, and order of treatment.
